# An Electron-Transporting Thiazole-Based Polymer Synthesized Through Direct (Hetero)Arylation Polymerization

**DOI:** 10.3390/molecules23061270

**Published:** 2018-05-25

**Authors:** Patricia Chávez, Ibrahim Bulut, Sadiara Fall, Olzhas A. Ibraikulov, Christos L. Chochos, Jérémy Bartringer, Thomas Heiser, Patrick Lévêque, Nicolas Leclerc

**Affiliations:** 1 ICPEES UMR 7515, Université de Strasbourg-CNRS, 25 rue Becquerel, Strasbourg 67087, France; patriciakirsch.chavez@gmail.com (P.C.); bulut.ciel@free.fr (I.B.); 2 ICube UMR 7357, Université de Strasbourg-CNRS, 23 rue du Loess, Strasbourg 67037, France; sadiara.fall@unistra.fr (S.F.); ibraikulov@unistra.fr (O.A.I.); j.bartringer@unistra.fr (J.B.); thomas.heiser@unistra.fr (T.H.); patrick.leveque@unistra.fr (P.L.); 3 Advent Technologies SA, Patras Science Park, Stadiou Street, Platani-Rio, Patra 26504, Greece; chochos@eie.gr

**Keywords:** direct (hetero)arylation polycondensation, *n*-type polymer, thiazole-based DPP, organic solar cell

## Abstract

In this work, a new *n*-type polymer based on a thiazole-diketopyrrolopyrrole unit has been synthesized through direct (hetero)arylation polycondensation. The molar mass has been optimized by systematic variation of the the monomer concentration. Optical and electrochemical properties have been studied. They clearly suggested that this polymer possess a high electron affinity together with a very interesting absorption band, making it a good non-fullerene acceptor candidate. As a consequence, its charge transport and photovoltaic properties in a blend with the usual P3HT electron-donating polymer have been investigated.

## 1. Introduction

Since the 1980s and the seminal work on light-emitting organic diodes by Tang and van Slyke at Kodak, the interest of the scientific community in organic semiconductors (OSCs) has been growing steadily [1,2]. Indeed, this work paved the way towards a new generation of optoelectronic devices: light, flexible, cheap, and therefore highly attractive. Since then, devices and OSC chemistry have continuously progressed. Organic light-emitting diodes (OLEDs) are now typical electronic components for the display applications market, and organic photovoltaic (OPV) devices exhibit power conversion efficiencies (PCE) as high as those of standard amorphous silicon-based devices [3,4].

However, despite these obvious progresses in OSC science, the development of *n*-type materials lags far behind that of their *p*-type counterparts. This is partly due to the intrinsic efficiency and versatility of the fullerene derivatives used as electron acceptor materials in many devices [5]. However, in the face of growing demands for highly absorbing *n*-type materials for OPV devices and given the growing diversification of OSC applications, including in thermoelectricity, new *n*-type materials have emerged recently [6]. Among them, imide- and amide-functionalized materials exhibit interesting physical and chemical properties, combining high extinction coefficients and high electron mobilities [7]. The diketopyrrolopyrrole (DPP) unit is a well-known prototype of such a functional group. Thanks to a high intrinsic electron affinity (EA), excellent thermal- and photostability [8], and a planar geometry favorable for π–π stacking interactions [9], it has been used as an electron-withdrawing unit in many OSCs. Together, these features make DPP-based materials promising candidates for high-performance organic field effect transistors (OFET) and OPV devices [10]. The DPP can be seen as a bis-lactam unit sandwiched by two aromatic substituents at the 3 and 6 positions. It has been clearly observed that the flanking aromatic substituents strongly modulate the optoelectronic properties. Thus, flanking thiophenes minimize steric hindrance on the DPP core and lead to coplanar thiophene-based DPP building blocks that are widely used in conjugated polymers. However, further modifications are needed to increase its electron affinity and promote the electron mobility. Thus, in 2013, Park et al. used a fluorinated phenylene in order to increase this EA [11]. By using an increasing level of fluorination, they clearly demonstrated that the lowest unoccupied molecular orbital (LUMO) energy level of the polymer becomes lower, and thereby, the charge-transport behavior in field-effect transistors changes from *p*-type to *n*-type. More recently, Bura et al. reported that the flanking fluorinated thiophene on a DPP core induces ambipolarity in OFETs [12]. Another strategy lies on the use of intrinsically more electronegative flanking aromatic units. In this way, Li et al. recently reported an impressive electron mobility of 6.3 cm^2^∙V^−1^∙s^−1^ for a polymer built from a 2-pyridinyl-substituted DPP block as the comonomer [13]. Following the same idea, our group and others reported the use of the thiazole unit as an aromatic flanking unit [14,15,16]. The presence of the electronegative nitrogen increases the electron affinity compared to a standard thiophene unit [17]. As a consequence, thiazole-flanked DPP-based polymers exhibit higher electron affinity than their thiophene counterparts [16,18].

However, thiazole-based DPP suffers from a major drawback from the chemical point of view that is often hidden in the literature. Indeed, its functionalization by halogens in accurate positions, allowing further polycondensation reactions, is very difficult and particularly ineffective, as in best cases the bromination step does not lead to a yield of more than a few percent [18,19]. Very often, the thiazole-based DPP molecule degrades during the bromination step (the solution becomes uncolored, probably due to lactam opening under acidic conditions). 

In this context, the direct (hetero)arylation polymerization (DHAP) appears to be a very promising and efficient synthetic tool, allowing skipping of these functionalization steps. Indeed, this reaction recently extended the scope of standard palladium-catalyzed cross-coupling to the sp^2^ C–H bond of a (hetero)aryl derivative with a (hetero)aryl halide as coupling partner [20]. As a consequence, it allows skipping of the organometallic derivative chemical step, in addition to a simplification of the purification steps and the absence of toxic organometallic byproducts [21]. However, the DHAP is not as simple and straightforward to implement as standard palladium-catalyzed cross-coupling polycondensations. In particular, the regioselectivity control, due to the numerous reactive C–H bonds present on organic molecules, appears as a major challenge that some groups have already addressed, thanks to theoretical calculations [22]. Another strategy consists of protecting the C–H bonds that should not react with groups of low steric hindrance, such as methyl groups [23]. 

This is the strategy that we followed herein, in order to obtain a thiazole-flanked DPP-based polymer obtained by DHAP. In order to stay as simple as possible, we selected the thiophene unit as the comonomer, protected in β-positions by methyl groups. A focus was placed on polymer molar mass optimization, thanks to monomer concentration variation. The optical properties and the frontier orbitals' energies have been characterized and discussed with regard to the molar mass evolution. Finally, we report the preliminary evaluation of their electronic properties, through studies of their application as organic field effect transistors and as non-fullerene acceptors (NFA) in organic solar cells.

## 2. Results

### 2.1. Synthesis and Characterization

**Scheme 1:**
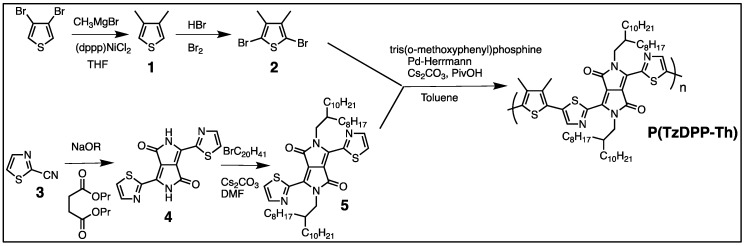
Synthesis of P(TzDPP-Th) by direct (hetero)arylation polymerization (DHAP). See materials and method section for experimental details.

The synthetic pathway is depicted in Scheme 1.

As discussed in the introduction, as the thiazole-based DPP is very difficult to halogenate, we decided to use the thiophene unit as the (hetero)aryl halide coupling partner. However, in order to avoid β-branching on the 3- and 4-positions of the thiophene unit, we protected them with small methyl groups [24,25]. One can notice that a similar polymer was recently synthesized by DHAP and published by Kuwabara et al. [26]. As we did in the present study, they protected the β-position, but by using an electron-rich 3,4-ethylylenedioxythiophene (EDOT) unit instead of a simple thiophene; but surprisingly, they used the dibrominated derivative of the thiazole-based DPP. The dibromo monomer (compound **2**) was synthesized through a two-step chemical route from the commercially available 3,4-dibromothiophene. The alkylated thiazole-based DPP (compound **5**) has been synthesised following the already published procedure [14]. Further details about monomer synthesis could be found in the materials and method section. As already noticed in some publications, the monomer concentration is a key factor governing the polymerization efficiency, and finally, the degree of polymerization (DPn) [22,27,28]. In line with this observation, using a simple and typical catalyst system (already developed for thiazole-based materials) [29], we varied the monomer concentration from 0.05 to 0.26 M. Accordingly and in agreement with previous reports, the molar mass of the P(TzDPP-Th) increases with the monomer concentration (see Table 1 and Figures S1–S8 in the Supplementary Materials).

By using monomer concentrations higher than 0.2 M, we finally succeeded to reach rather reasonable molar masses, on average of about 56 kg/mol (measured in chlorobenzene (CB) at 80 °C).

**Figure 1:**
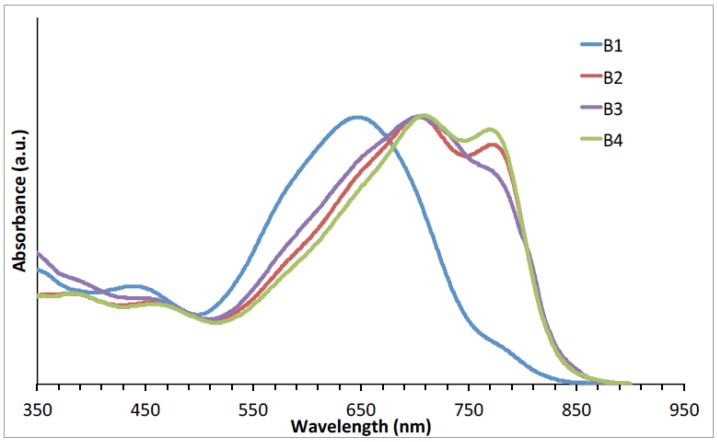
UV–vis absorption spectra in *orto*-dichlorobenzene solution of all P(TzDPP-Th) batches (B1–B4).

**Figure 2:**
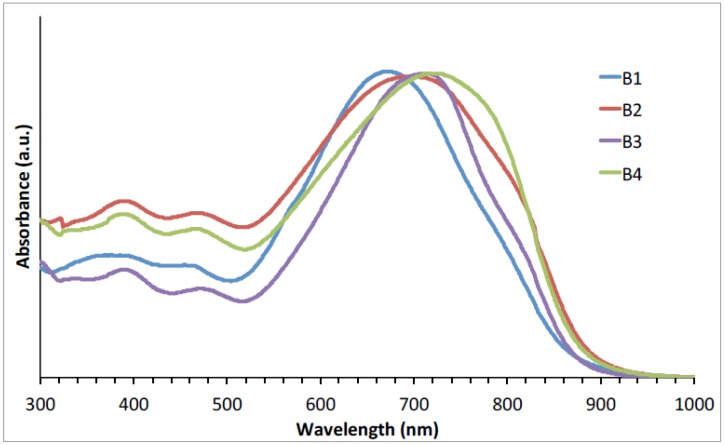
UV–vis absorption spectra in thin film of all P(TzDPP-Th) batches.

The UV–vis spectra of all fractions have been recorded in *orto*-dichlorobenzene (*o*-DCB) solution (Figure 1) as well as in thin film (Figure 2). Interestingly, optical features are significantly different and display a molar mass dependence. Thus, in solution, the low B1 batch exhibits a hypsochromic-shifted broad band centered around 640 nm, while the two higher molar mass batches show two-peak characteristic broad bands with maxima at approximately 700 and 770 nm.

In the solid state (Figure 2), similar trends are observable, despite smaller differences in maximum absorption wavelengths. A lower molar mass leads to a significant hypsochromic shift with regard to higher molar masses. The highest molar mass exhibits an even bathochromic shift with the absorption maximum at 719 nm. 

Such behaviour is rather consistent with literature data and means that the effective conjugation length is reached for a DPn higher than 5.

**Figure 3:**
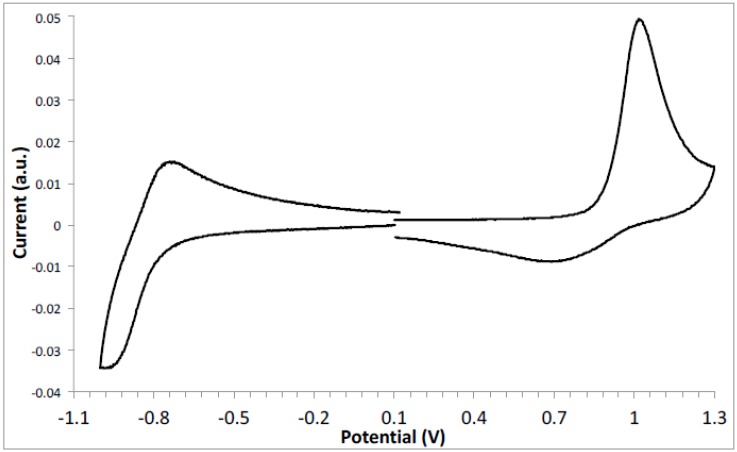
Cyclic voltammogram of P(TzDPP-Th) in thin film recorded in acetonitrile 0.2 M [NBu_4_][PF_6_]. Platinum working electrode sweep-rate: 100 mV∙s^−1^.

Cyclic voltammetry (CV) of the polymers was carried out in a three-electrode cell using an Ag/AgCl reference electrode and calibration with the ferrocene/ferrocenium (Fc/Fc+) redox couple. The cyclic voltammetry curves are shown in Figure 3 and Figure S9 in the Supplementary Materials. No differences could be observed from CV with regard to the molar mass values. All batches exhibit a quasi-reversible reduction peak at around −0.7 V (E_red_), while a quasi-reversible oxidation peak is recorded at 0.8 V (E_ox_). From the ionisation potential and the electronic affinity measured by CV, the corresponding highest occupied molecular orbital (HOMO) and LUMO energy levels (E_HOMO_ and E_LUMO_, respectively) were calculated using the following equations: E_HOMO_ = −(E_OX_ + 4.8) = −5.6 eV and E_LUMO_ = −(E_RED_ + 4.8) = −4.1 eV.

**Figure 4:**
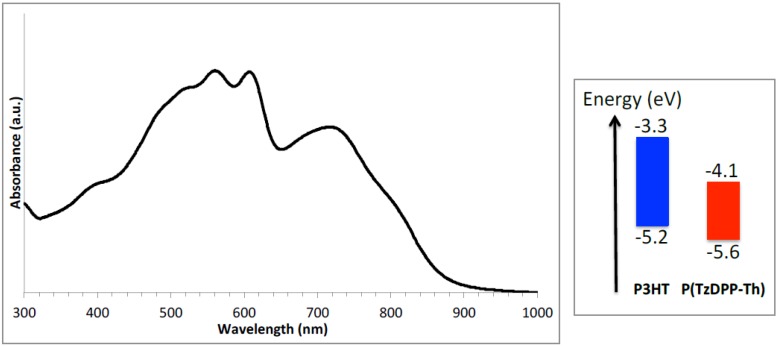
UV–vis spectrum of a P3HT/P(TzDPP-Th) blend (mass ratio 1/1) in thin film (**left**); energy level diagram of P3HT and P(TzDPP-Th) (**right**).

The P(TzDPP-Th) exhibits a rather high electron affinity that makes it a possible candidate to be used as an electron-transporting material and potentially as a non-fullerene acceptor (NFA) in organic solar cells. In addition, its energy levels are accurately positioned to be used as an electron-accepting material for the well-known high band-gap electron-donating poly(3-hexylthiophene) (P3HT) (see Figure 4, right). Moreover, due to its low band-gap nature, this polymer is highly complementary to the absorption of P3HT, as only a very small overlap could be seen around 600 nm (see Figure 4, left).

**Figure 5:**
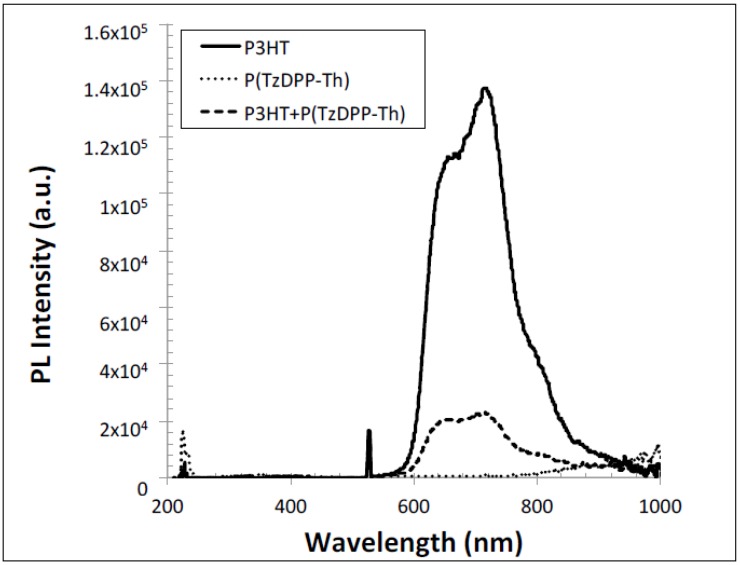
photoluminescence spectra for a pure P3HT film, a pure P(TzDPP-Th) film, and a P3HT/P(TzDPP-Th) film with a mass ratio of 1/1. All films were deposited by spin-coating from *o-*DCB solutions (3 mg/mL) on glass substrates with a film thickness of (100 ± 6) nm. The excitation wavelength was 532 nm.

Further, photoluminescence (PL) spectra were recorded on different films using an excitation wavelength of 532 nm. As shown in Figure 5, the PL spectrum of P3HT is very efficiently quenched when P3HT is blended with P(TzDPP-Th) at a mass ratio of 1/1. As a consequence, we elaborated organic photovoltaic devices from a P3HT/P(TzDPP-Th) blend.

### 2.2. Electronic Properties

Organic photovoltaic (OPV) devices were elaborated using both standard (glass/ITO/PEDOT: PSS/active layer/Ca/Al) and inverted (glass/ITO/PEIE/active layer/MoO_3_/Ag) architectures. The batch B1 has been intentionally put aside due to its poor film ability and the low expectation coming from its very low molar mass. The ratios of P3HT:P(TzDPP-Th) (called D:A ratio in Table 2) and total solution concentrations were varied from 1:0.5 to 1:2 and between 4.5 mg/mL and 9 mg/mL, respectively. Also, for device optimization, active layers were spin-coated from different solvents (*o*-DCB and CHCl_3_). Acceptable film qualities were obtained from CHCl_3_ solutions. For all batches, the best conditions were found to be 1:0.5 and 4.5 mg/mL as the total polymer concentration in CHCl_3_ as the active layer in an inverted structure. Table 2 summarizes the best photovoltaic parameters obtained. 

**Figure 6:**
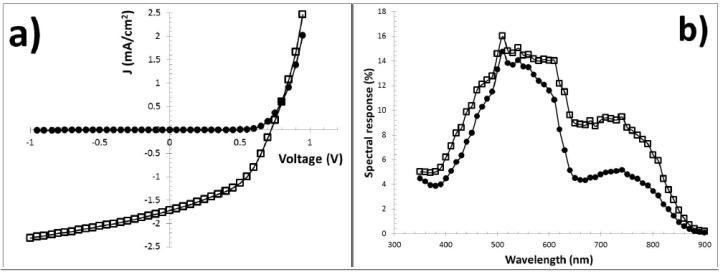
(**a**) current density versus voltage measurements obtained for an inverted solar cell using a P3HT/P(TzDPP-Th) blend (mass ratio 1/0.5) as the active layer in the dark (closed symbols) and under standard AM 1.5 G 100 mW/cm^2^ illumination conditions (open symbols). The corresponding external quantum efficiency (closed symbols) and internal quantum efficiency (open symbols) spectra are displayed in (**b**).

In optimized conditions, the best photovoltaic efficiencies (PCE ≈ 0.6%) were recorded for the batch B2 using the inverted architecture. The corresponding current density versus voltage (J–V) characteristics in the dark and under standard illumination conditions (AM 1.5 G, 100 mW/cm^2^) are reported in Figure 6 together with the external quantum efficiency (EQE) measured for the same cell.

The strong variation of J as a function of V in the reverse polarization direction (Figure 6a) is a strong indication of poor charge extraction. In the spectral response (Figure 6b), we can clearly distinguish the contribution from P3HT (from roughly 400 to 600 nm) from the one from P(TzDPP-Th) (from roughly 600 to 850 nm). The contribution from P3HT to the EQE is much more pronounced than the one from P(TzDPP-Th), even in such a P3HT-rich blend. The internal quantum efficiency (IQE) is more in line with the relative P3HT/P(TzDPP-Th) mass ratio, which could be an indication of a rather poor P(TzDPP-Th) absorption coefficient. When we go from the lower Mn to the higher one (from B2 to B4), we clearly observe a decrease of the open-circuit voltage (V_oc_) and of the short-circuit current density (J_sc_) of the solar cells. This trend is rather counterintuitive if we consider that in general, the charge transport properties are improved when the polymer molar mass is increased. However, it may suggest that higher Mn batches could have a lower miscibility with P3HT, that in turn, could lead to macrophase separation. The lower solubility of the higher molar mass batches supports this hypothesis. In order to understand further the relatively low PCE of our solar cells, the electron mobilities (µ_e_) of the polymers were evaluated by using organic field effect transistors in standard bottom-gate top-contact configuration (see Section 3). The extracted mobilities from the saturation regime are summarized in Table 2 (see Figures S10 and S11 in Supplementary Materials for OFET transfer curves). As expected, the molar mass increase leads to a slight increase of the electron mobility. Only the very low-molar-mass B1 batch does not show any field effect. However, all batches exhibit µ_e_ values of approximately 10^−5^–10^−4^ cm^2^∙V^−1^∙s^−1^, which are low compared to µ_e_ in pure PC_60_BM (10^−2^∙cm²∙V^−1^∙s^−1^). These electron mobilities can become even lower in P3HT:P(TzDPP-Th) blends, leading to a high charge recombination rate and poor charge extraction, as seen even in the characteristics of the best photovoltaic devices. This might partly explain the moderate PCEs obtained in solar cells, despite the improved photon harvesting capabilities as compared to P3HT/PC_60_BM blends. 

## 3. Materials and Methods 

### 3.1. Materials

All reagents and chemicals were purchased commercial sources. THF, Et_2_O, and toluene were distilled from sodium. Tris (*o*-methoxyphenyl)phosphine and other reagents and chemicals were used as received. *trans*-di(μ-acetato)bis[*o*-(di-*o*-tolyl-phosphino)benzyl]dipalladium (II), thiazole-based DPP (compound **4**), and 2,5-dibromo-3,4-dimethylthiophene were prepared as described in the literature [14,30,31]. Chromatographic purifications were performed using 40–63 μm silica gel. Thin layer chromatography was performed on silica gel plates coated with fluorescent indicator.

### 3.2. Characterizations

NMR spectroscopy: The 300, 400 (^1^H) and 75, 100 MHz (^13^C) NMR spectra were recorded at room temperature using perdeuterated solvents as internal standards on a Bruker Advance spectrometer (Strasbourg, France). 

Gel permeation chromatography (GPC) (Athens, Greece): Average molecular weights per number (Mn) and polydispersity indices (*Đ*) were determined by GPC at 80 °C on a Shimadzu liquid chromatograph (LC-20AD) system consisting of a DGU-20A5R degassing unit, a SIL-20AC HT auto sampler, a CTO-20AC column oven, a SPD-20AV UV–vis detector, and a RID-20A refractive index detector connected in series. The system contains a PL-GEL 10 μm guard column, two PL-GEL 10 μm Mixed-B columns, and chlorobenzene (CB) as the eluent. The instrument was calibrated with narrow polystyrene standards with M_p_ ranging from 4730 to 3,187,000 g/mol. 

UV–visible spectroscopy (Strasbourg, France): Absorption spectra in solution and in thin films were recorded on a Shimadzu UV-2600 spectrophotometer. In the solid state, the absorption spectra were measured on thin films drop-casted on glass substrates from a 1 mg/mL *o*-DCB solution of polymers. 

DSC measurements (Strasbourg, France): DSC measurements were performed with a TA Instruments Q1000 instrument, operated at a scanning rate of 5 °C min^−1^ on heating and on cooling. 

Electrochemical measurements (Strasbourg, France): Oxidation and reduction potentials were determined by cyclic voltammetry with a conventional 3-electrode system using a voltammetric analyzer equipped with a platinum microdisk (2 mm^2^) working electrode and a platinum wire counter electrode. Potentials were calibrated versus the saturated calomel electrode (SCE), using the ferrocene/ferricinium couple as an internal reference (+0.38 V vs SCE) and a conventional scan rate of 200 mV/s. Recrystallized tetrabutylammonium hexafluorophosphate (Bu_4_NPF_6_) was used as the supporting electrolyte (0.1 M) in distilled and anhydrous acetonitrile. Acetonitrile was distilled from CaH_2_ under a nitrogen atmosphere. 

Photoluminescence measurements (Strasbourg, France): Photoluminescence measurements using a SPEX Fluorolog-2 spectrofluorometer were carried out on films deposited on top of a glass substrate with an excitation wavelength of 532 nm. Pure polymers and blends were spin-coated from a 3 mg/mL *o*-DCB solution, and the film thickness measured by profilometry was in the (100 ± 6) nm range for every film,

### 3.3. Synthesis

*Synthesis of 2,5-bis-(2-octyl-dodecyl)-3,6-bis-thiazol-2-yl-2,5-dihydro-pyrrolo[3,4-c]pyrrole-1,4-dione* (**5**). 1.5 g (5 mmol) of compound **4** were introduced in a dry, degassed 2-neck round-bottom flask filled with argon. To this were added 50 mL of anhydrous DMF and 4.07 g (12.5 mmol) of Cs_2_CO_3_. The reaction mixture was stirred at 60 °C for 1 h. The reaction mixture was then cooled to room temperature and 12.5 g (35 mmol) of 2-octyldodecyl bromide was added dropwise and the temperature was increased to 100 °C overnight. The mixture was then cooled to room temperature and organic compound was extracted with diethyl ether and washed with water several times. The organic solution was dried over sodium sulfate, concentrated, and purified by silica gel chromatography with a mixture of methylene chloride and petroleum ether (80/20) as eluent to yield the title compound as purple solid (2 g, 46%). ^1^H-NMR (300 MHz, CDCl_3_) δ: 8.05 (*d*, 2H, ^3^*J* = 3.1 Hz), 7.69 (*d*, 2H, ^3^*J* = 3.1 Hz), 4.34 (*d*, 4H), 1.86 (m, 2H), 1.2–1.3 (m, 64H), 0.86 (m, 12H); ^13^C-NMR (75 MHz, CDCl_3_) δ: 161.2, 155.3, 144.2, 138.0, 123.8, 110.5, 46.8, 37.9, 31.93, 31.90, 31.4, 30.0, 29.7, 29.64, 29.60, 29.5, 29.4, 29.35, 29.30, 26.4, 22.7, 14.11.

*Polymerization of P(TzDPP-Th)*. A flame-dried Schlenck was charged with brominated monomer, compound **2** (1.0 equiv.), Pd-Herrmann (0.08 equiv.), tris(*o*-methoxyphenyl)phosphine (0.16 equiv.), Cs_2_CO_3_ (2.2 equiv.), and PivOH (0.4 equiv.). Dried and degassed toluene (at various concentrations, see Table 1) was added under inert gas followed by compound **5** (1.0 equiv.) and the mixture was stirred for 48 h at 120 °C. Then, the crude polymer was purified by precipitation in methanol, filtered, and separated by Soxhlet extraction with methanol, acetone, cyclohexane, and chlorobenzene. Then, the sodium diethyldithiocarbamate solution was added to the chlorobenzene fraction and the mixture was stirred at 60 °C for 1 h. The organic phase was washed with water, separated, and evaporated under reduced pressure. Finally, the polymer was precipitated in methanol, filtered, and dried under reduced pressure at 40 °C overnight, providing a powder with a metallic shine. ^1^H-NMR (400 MHz, CDCl_3_) δ: 8.0 (m, 2H), 4.2 (s, 4H), 2.3 (m, 6H), 1.9 (s, 2H), 1.2 (m, 64H), 0.8 (m, 12H).

### 3.4. Organic Device Elaboration

Field effect mobility measurements: Bottom-gate top-contact organic field-effect transistors (OFETs) were constructed. A 230 nm thick silicon oxide was used as the gate dielectric, and an *n*-doped (3 × 10^17^/cm^3^) silicon crystal as the gate electrode. The substrates were cleaned in acetone and isopropyl alcohol and subsequently in an ultraviolet ozone system for 30 min. Then, hexamethyldisilazane (HMDS) was spin-coated (500 rpm for 5 s and then 4000 rpm for 50 s) under nitrogen ambient atmosphere followed by an annealing step at 130 °C for 10 min. 5 mg/mL anhydrous chloroform of polymer solutions were spin-coated (1250 rpm for 60 s and 2250 rpm for 60 s). To complete the OFET devices, 20 nm of calcium and 120 nm aluminium top electrode were evaporated. The samples were then left overnight under vacuum (<10^−6^ mbar) to remove residual solvent traces. Both the OFET elaboration and characterizations were performed in nitrogen ambient atmosphere. The transistor output and transfer characteristics were recorded using a Keithley 4200 semiconductor characterization system. The charge carrier mobility was extracted in the saturation regime using the usual formalism on field effect transistor devices annealed at the same temperature as the optimized photovoltaic devices. 

Photovoltaic devices elaboration and characterization: for the inverted structure devices, ITO-coated glass was utilized as a substrate. A thin polyethyleneimine ethoxylated (PEIE) layer (<10 nm) was spin-coated onto precleaned ITO and thermally annealed at 100 °C for 10 min and used as an electron-extracting electrode. Active layers were constructed from *o*-DCB or CHCl_3_ solutions using blends of P3HT and P(TzDPP-Th) at mass ratios varying from of 1:0.5 to 1:2. Top electrode consisting of MoO_3_ (7 nm)/Ag (120 nm) was thermally evaporated under 5 × 10^−7^ mbar vacuum. For the direct structure devices, the very same active-layer solutions were deposited on ITO-coated glass on top of which PEDOT:PSS was already spin-coated (40 nm). For direct structure devices, the top electrode consisted of a Ca (20 nm)/Al (120 nm) bilayer. For both device structures, four diodes with a 12 mm^2^ active area were used per substrate. All the characterizations were done in nitrogen atmosphere under dark and simulated AM 1.5 G irradiation (100 mW/cm^2^, Lot Oriel Sun 3000 solar simulator).

The device spectral response was measured inside the glove-box using a home-made setup comprised of an Oriel 150 W solar simulator, a Jobin Yvon microHR monochromator (5 nm resolution), and a calibrated Si photodiode fitted after a beam-splitter to measure the incident light power for each wavelength band.

## 4. Conclusions

In summary, we succeeded in synthesizing by DHAP a thiazole-flanked diketopyrrolopyrrole-based polymer that exhibited a marked electron-transporting behaviour. By experimenting with the monomer concentration during the polymerization, various molar masses ranging from 4 kDa to 32 kDa have been obtained. Optical properties have shown a molar mass dependence, highlighting that an efficient conjugation seems to be reached with a minimum DPn of approximately 5 (around 5–6 kDa). The accurate electron affinity and absorption property of this P(TzDPP-Th) polymer make it a good candidate *n*-type material for photovoltaic devices. However, despite these positive features, photovoltaic devices using the P(TzDPP-Th) polymer in a blend with the usual P3HT exhibit moderate PCEs. This is due partly to the rather low electron mobility of the P(TzDPP-Th), consequently hindering the charge extraction ability of the active blend [32]. However, this first polymer prototype exhibits very encouraging optoelectronic properties. As a consequence, we strongly believe that after a few structural modifications, this family of polymers, created using DHAP, could be considered to be promising candidates for electron-transporting materials in optoelectronic devices.

## Figures and Tables

**Table 1:** Molecular weight characteristics and optical properties of the different P(TzDPP-Th) batches.

Polymer	Monomer (M)	(M_n_) (kg/mol)	(M_W_) (kg/mol)	Đ	Solution λ_max_ (nm)	Film λ_max_ (nm)	λ_onset_ (nm)	E_g_^opt^ (eV)
B1	0.05	2.7	5.8	2.2	643	665	875	1.42
B2	0.10	5.1	13.3	2.6	700,770	706	872	1.42
B3	0.15	7.6	20.5	2.7	699,781	692	905	1.37
B4	0.26	18.3	56.7	3.1	704,765	719	900	1.38

M_n_: number average molecular weight; M_W_: weight average molecular weight; Đ: polydispersity index, λ_max_: wavelength at which the highest signal (peak) is recorded; λ_onset_: maximum recorded wavelength; E_g_^opt^: optical band-gap.

**Table 2:** Photovoltaic (AM 1.5 G 100 mW/cm^2^ illumination conditions) and electronic properties of P(TzDPP-Th) batches spin-coated from CHCl_3_.

Polymer	µ_e_ (cm^2^∙V^−1^∙s^−1^)	Ratio D:A	Device Architecture	V_oc_ (*V*)	J_sc_ (mA∙cm^−2^)	FF (*%*)	PCE (%)
B2	2 × 10^−5^	1:0.5	inverted	0.72	1.72	46.3	0.58
B3	2 × 10^−5^	1:0.5	inverted	0.67	1.02	40.3	0.28
B4	8 × 10^−5^	1:0.5	inverted	0.65	0.77	40.2	0.20

µ_e_: electron mobility; V_oc_: open circuit voltage; J_sc_: short circuit current density; FF: fill factor; PCE: power conversion efficiency.

## Supplementary Materials

The following are available online, NMR traces Figures S1–S4: SEC chromatograms Figures S5–S8, Cyclic voltammogram of ferrocene vs saturated calomel electrode Figure S9 and OFET output and transfer characteristic Figures S10–S11.
