# Supplementary material for: An Electron-Transporting Thiazole-Based Polymer Synthesized Through Direct (Hetero)Arylation Polymerization
Source: Molecules. 2018 May 25;23(6):1270. doi: 10.3390/molecules23061270 (PMC6100489; doi:10.3390/molecules23061270)
Supplement: Supplementary file 1 [file molecules-23-01270-s001.pdf]

Electron-transporting Thiazole-based polymer synthesized through direct (hetero)arylation polymerization

Patricia Chávez[1], Ibrahim Bulut[1], Sadiara Fall[2], Olzhas Ibraikulov[2], Christos L. Chochos[3], Jérémy Bartringer[2], Thomas Heiser[2], Patrick Lévêque[2] and Nicolas Leclerc[1,*]

[1] ICPEES UMR 7515, Université de Strasbourg-CNRS, 25 rue Becquerel, 67087 Strasbourg, France; leclercn@unistra.fr
[2] ICube UMR 7357, Université de Strasbourg-CNRS, 23 rue du Loess, 67037 Strasbourg, France; patrick.leveque@unistra.fr
[3] Advent Technologies SA, Patras Science Park, Stadiou Street, Platani-Rio, 26504, Patra, Greece; chochos@eie.gr
*Correspondence: leclercn@unistra.fr; Tel.: +33-368-852-709

Supplementary Information.

1. Polymer ^{1}H NMR traces

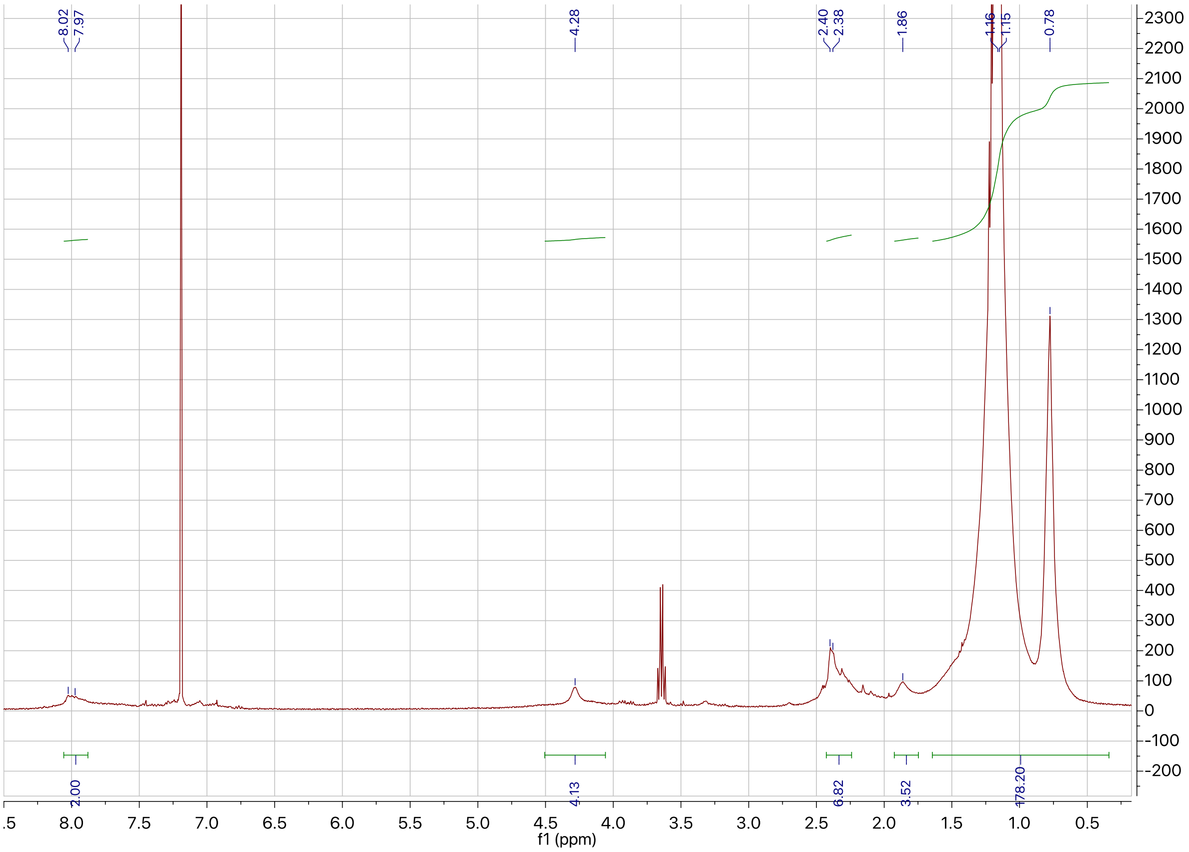

Figure S1. ^{1}H NMR spectrum of the P(TzDPP-Th) batch B1 in $CDCl_3$

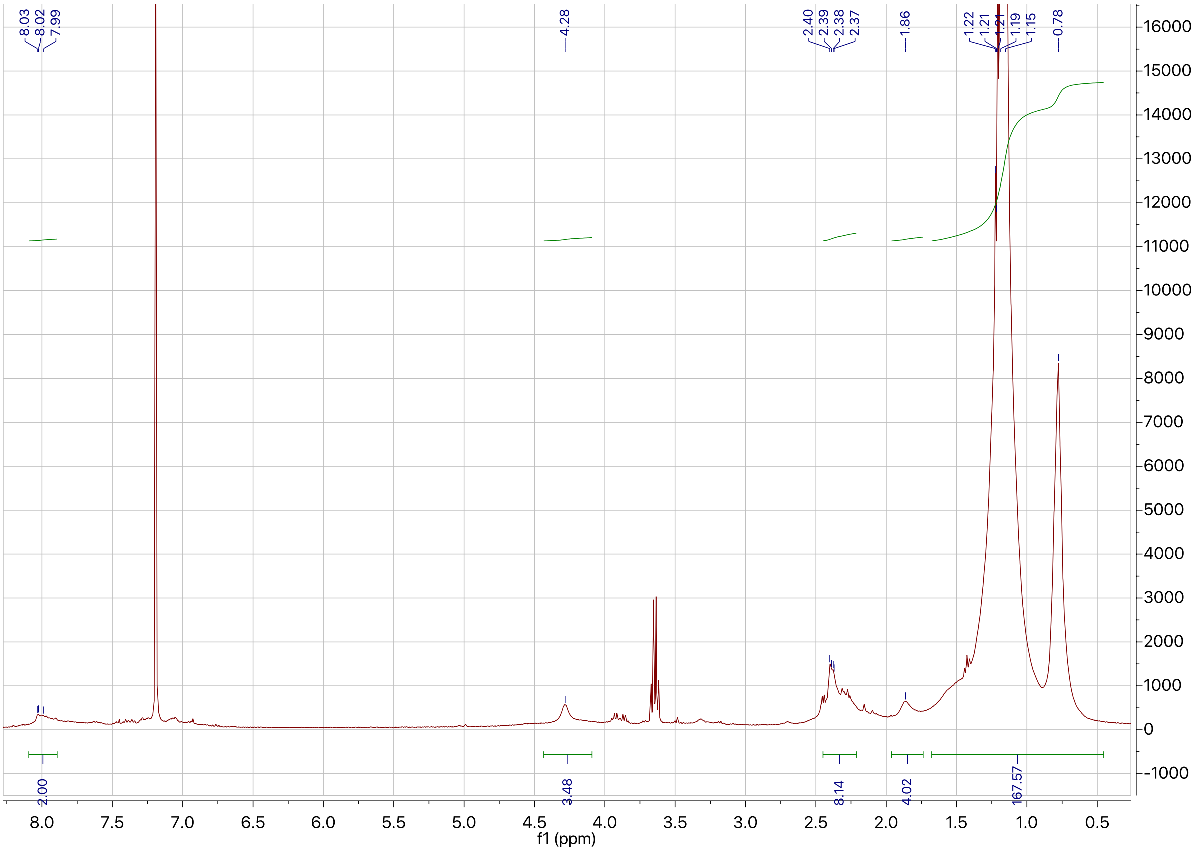

Figure S2. ^{1}H NMR spectrum of the P(TzDPP-Th) batch B2 in $CDCl_3$

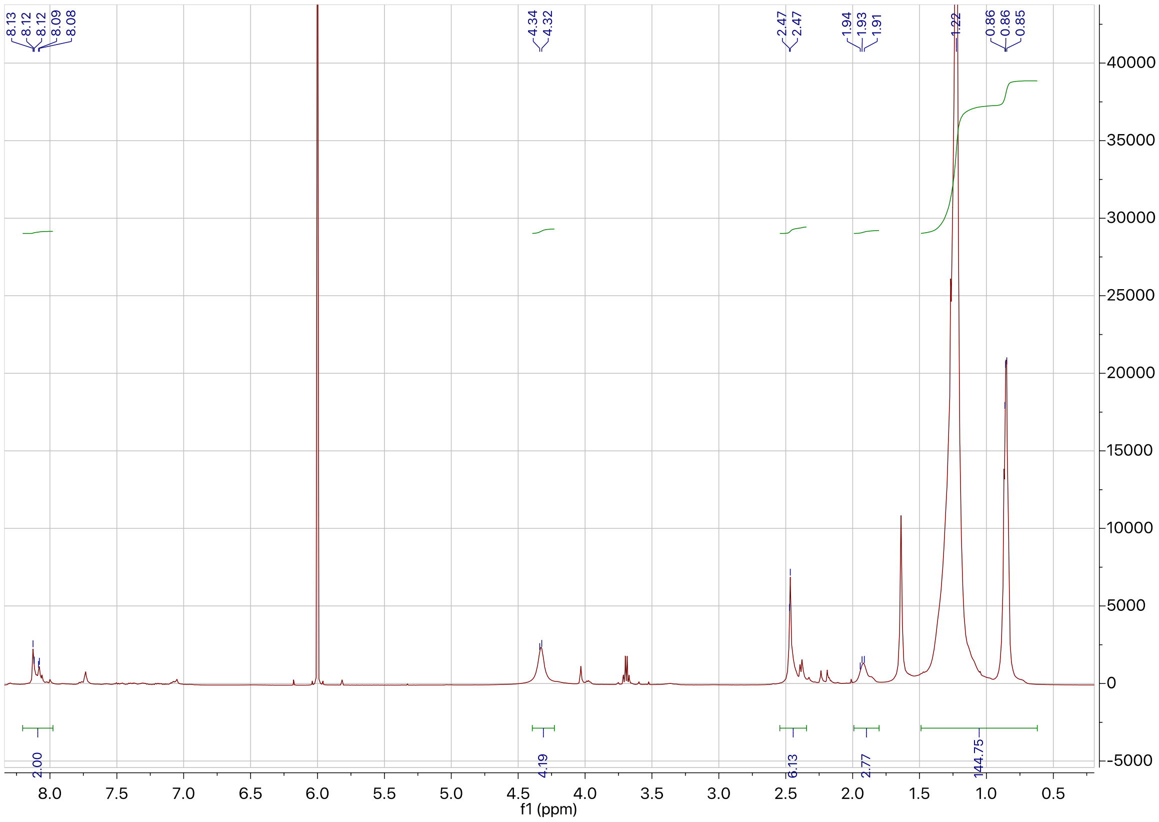

Figure S3. ^{1}H NMR spectrum of the P(TzDPP-Th) batch B3 in $C_2D_2Cl_4$

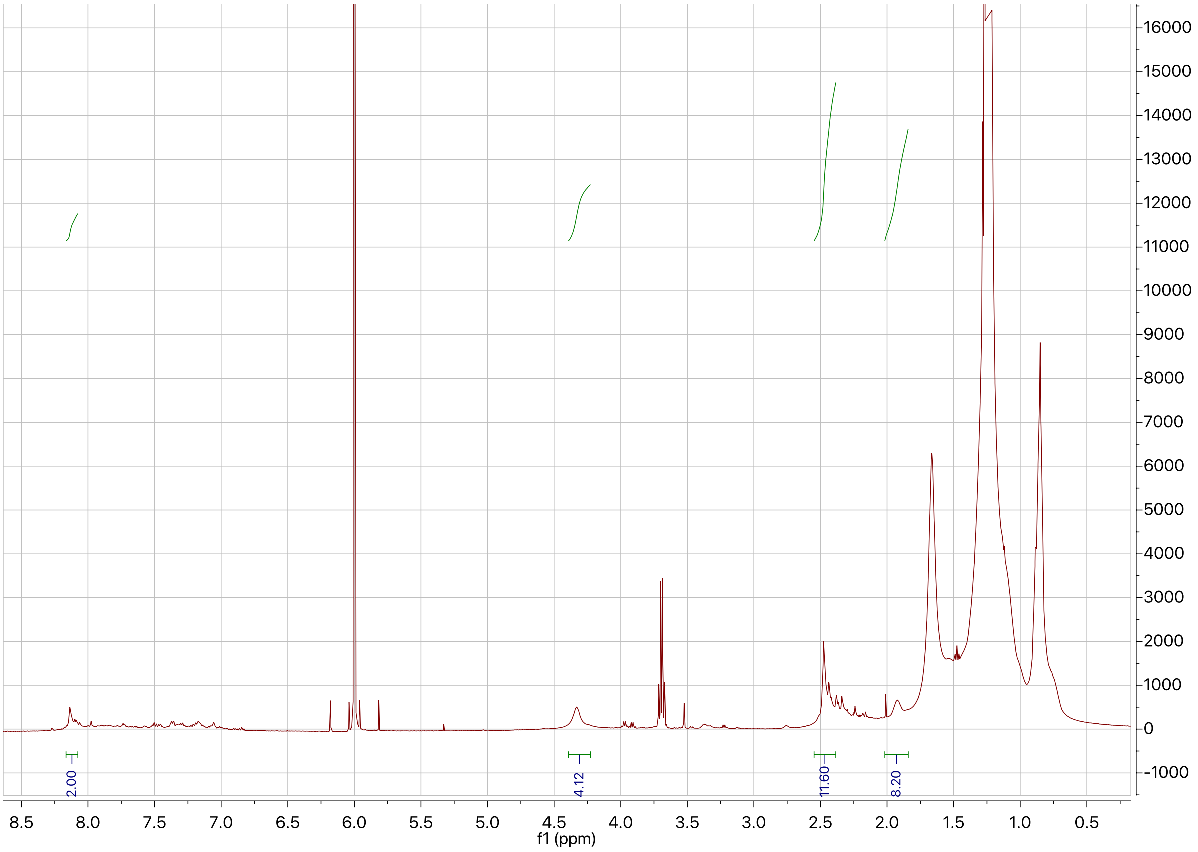

Figure S4. ^{1}H NMR spectrum of the P(TzDPP-Th) batch B4 in $C_2D_2Cl_4$

2. Polymer SEC chromatograms

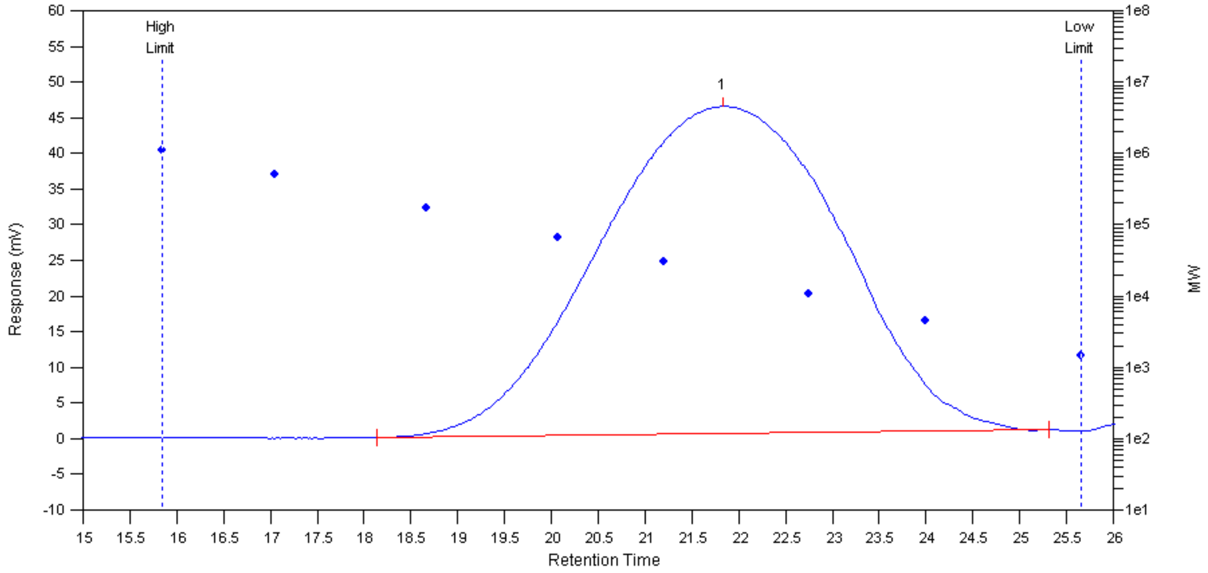

Figure S5. SEC chromatogram of the P(TzDPP-Th) batch B1

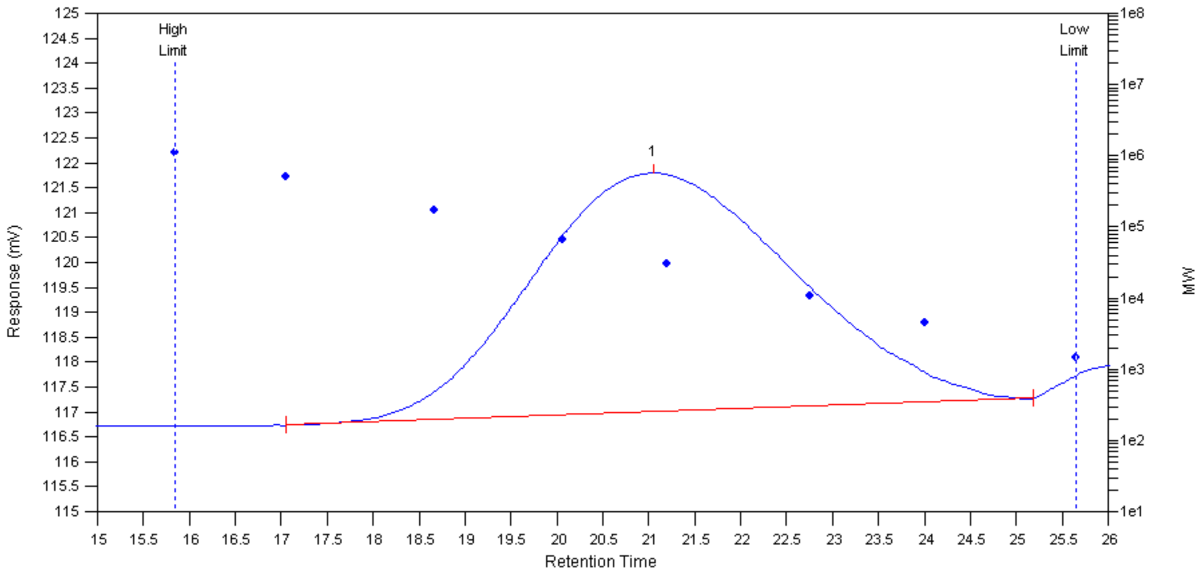

Figure S6. SEC chromatogram of the P(TzDPP-Th) batch B2

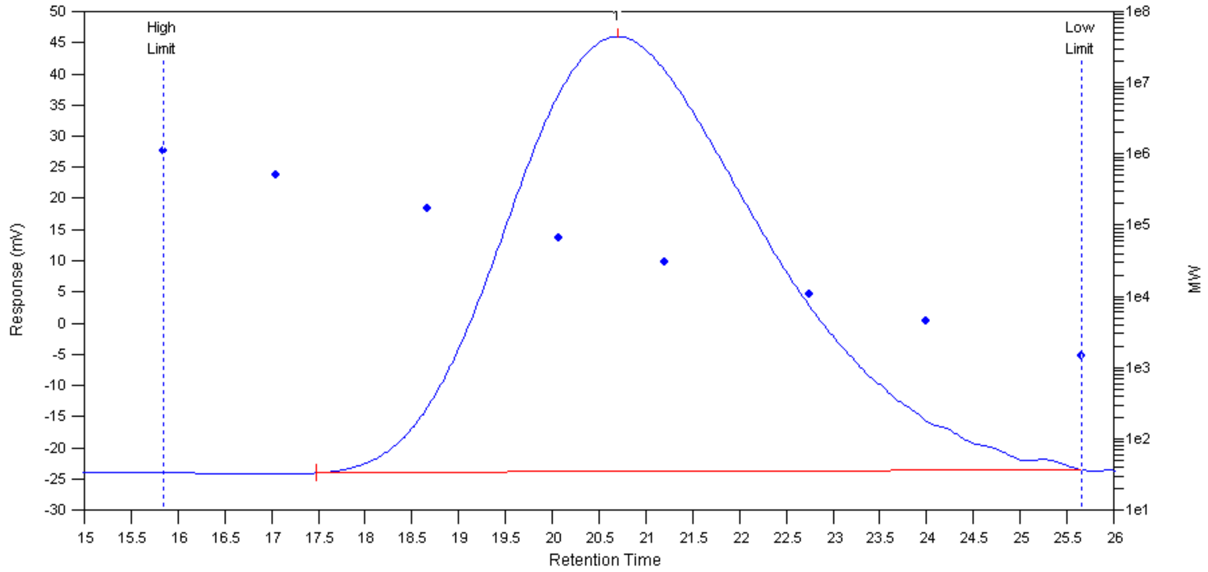

Figure S7. SEC chromatogram of the P(TzDPP-Th) batch B3

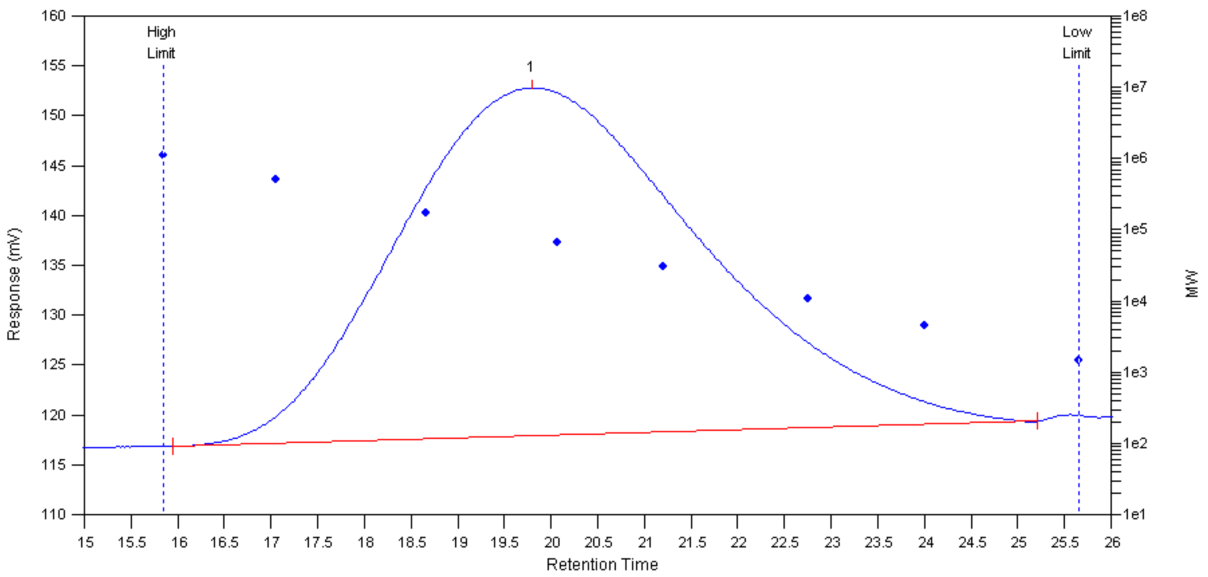

Figure S8. SEC chromatogram of the P(TzDPP-Th) batch B4

3. Cyclic voltammogram

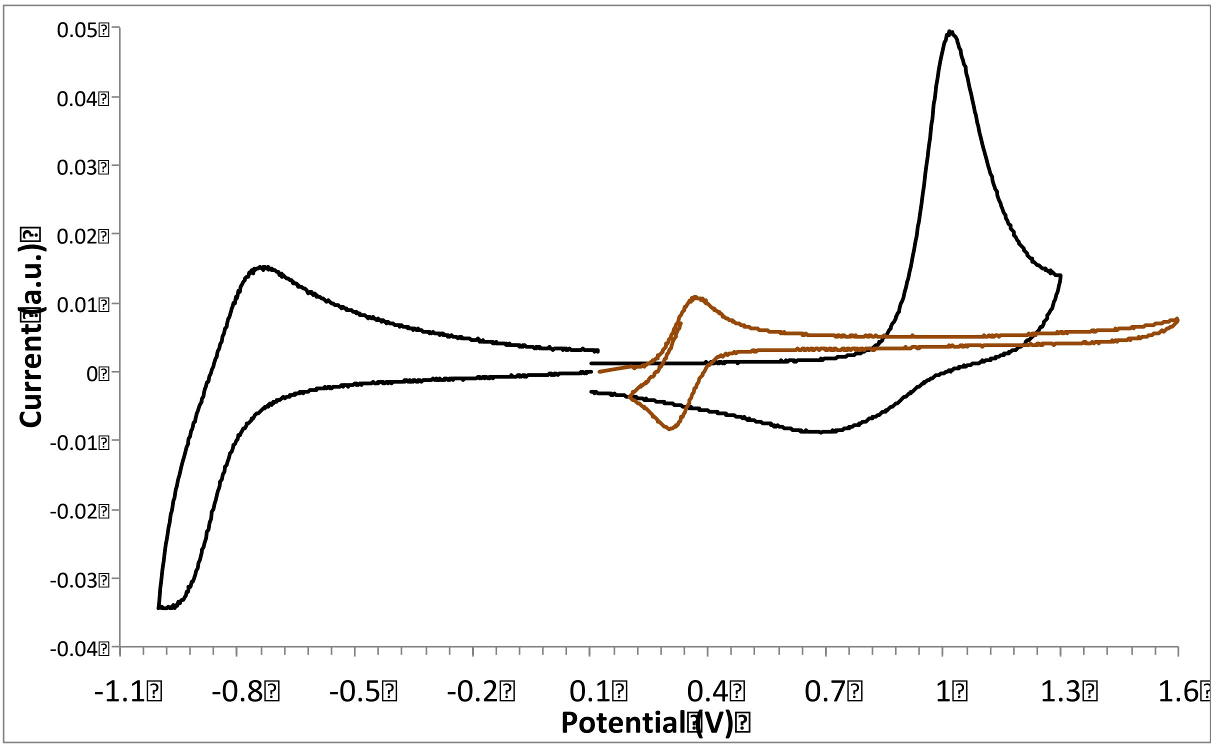

Figure S9. Cyclic voltammogram of P(TzDPP-Th) reference in thin-film recorded in acetonitrile + 0.2 M [NBu_4][PF_6]. Cyclic voltammogram of ferrocene vs SCE. Platinum working electrode, sweep-rate: 100 mV.s^{-1}.

4. OFET output and transfer characteristic

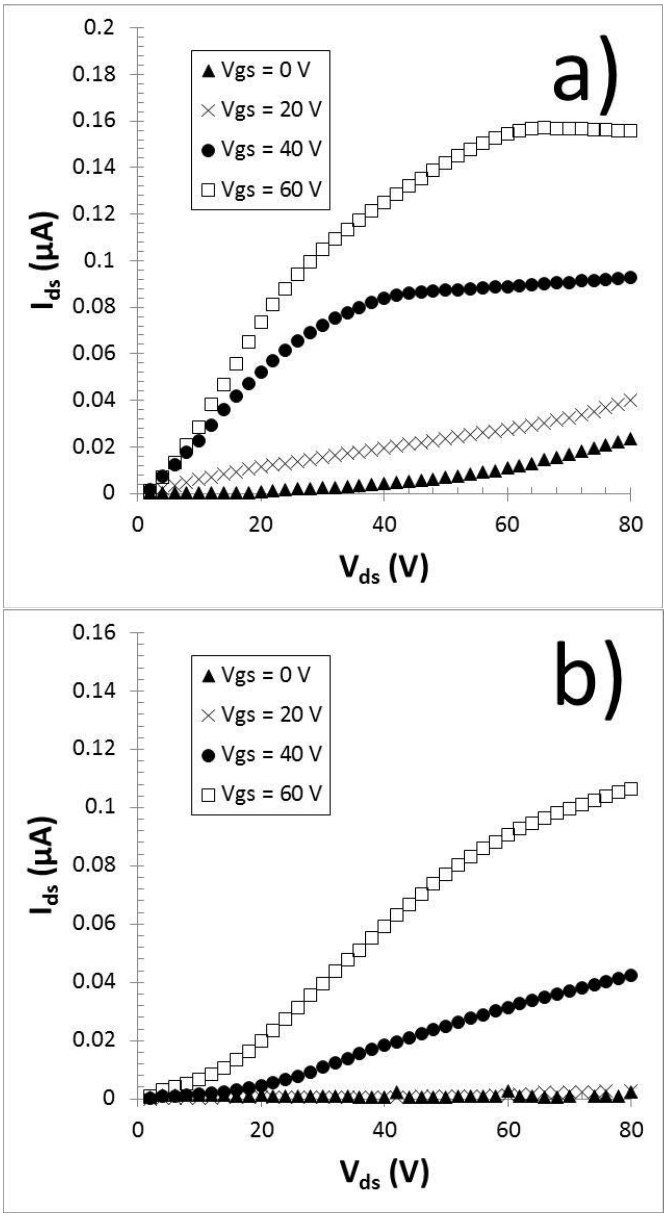

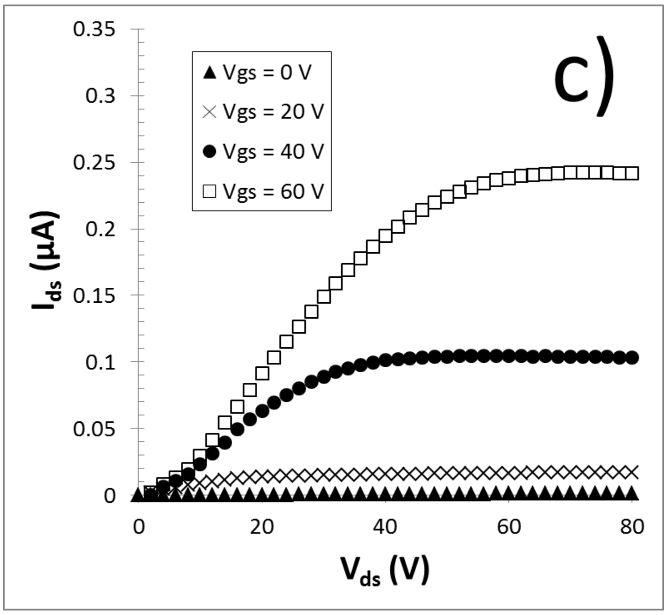

Figure **S10**. Output characteristics of the OFETs whose channel is **B2** (a), **B3** (b) and **B4** (c). I_{ds} is the drain-source current, V_{ds} the voltage difference between drain and source and V_{gs} the voltage difference between gate and source.

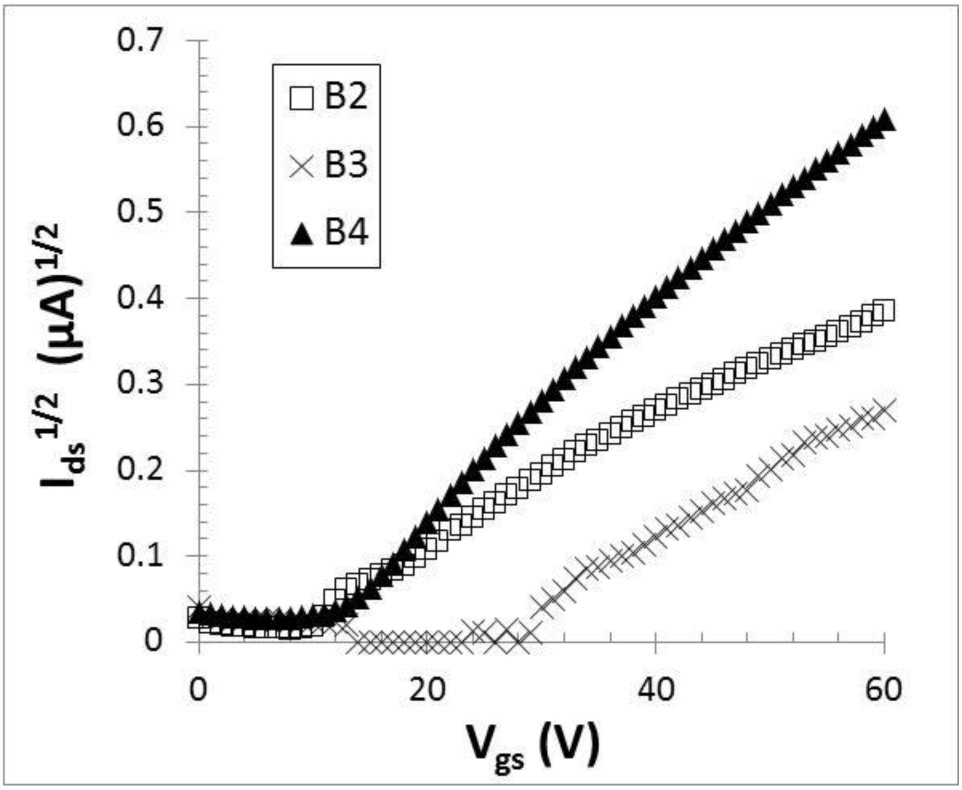

Figure **S11**. Transfer characteristics measured on the same **B2, B3** and **B4** OFETs at a drain-source voltage difference (V_{ds}) of 80 V. The electron mobility.is directly proportional to the slope of the square-root of I_{ds} as a function of V_{gs} using the standard formalism of OFETs in the saturation regime.
